# Cefepime Efficacy and Safety in Children: A Systematic Review and Meta-analysis

**DOI:** 10.3389/fped.2018.00046

**Published:** 2018-03-06

**Authors:** Saber Jan, Braveen Ragunanthan, Sandra R. DiBrito, Omolabake Alabi, Maria Gutierrez

**Affiliations:** ^1^Division of Pediatric Neurology, Department of Pediatrics The Hospital for Sick Children, Toronto, ON, Canada; ^2^Department of Pediatric, Taibah University, Medina, Saudi Arabia; ^3^Children’s Hospital of Pittsburgh of UPMC, Pittsburgh, PA, United States; ^4^Department of Surgery, Johns Hopkins Hospital, Baltimore, MD, United States; ^5^St. Francois Medical Centre, Abuja, Nigeria; ^6^Division of Pediatric Allergy and Immunology, Johns Hopkins University School of Medicine, Baltimore, MD, United States

**Keywords:** efficacy, safety, cefepime, children, antibiotic, cephalosporin

## Abstract

**Background:**

Cefepime is a fourth-generation cephalosporin antibiotic used to treat a variety of infections. The US Food and Drug Administration approved its use in certain types of infections among pediatric patients, and yet there have been mixed data about its efficacy and safety in this population.

**Objective:**

The objective of this review is to compare efficacy and all-cause mortality of cefepime to other clinically indicated antibiotics among children.

**Methods:**

We conducted a systematic search of MEDLINE, EMBASE, CENTRAL, LILACS, and clinicaltrials.gov databases through February 8, 2016. We included randomized controlled trials comparing cefepime to other clinical antibiotics, placebo, or no treatment in children aged 0–19 years in the inpatient setting with clinical signs of infection. The primary outcome of interest was all-cause mortality. The secondary outcomes were success rate, treatment failure, and incidence of adverse events. Study quality was assessed using the Cochrane Risk of Bias Assessment Tool.

**Results:**

Seventeen studies met the inclusion criteria. There was a total of 1,285 participants included, 624 participants in the cefepime arm and 661 in the comparison arm. A random effects meta-analysis for all-cause mortality showed no difference in rates of mortality between cefepime and comparator antibiotics with a mortality risk ratio of 0.88 (95% CI: 0.71–1.08). For the secondary outcomes of success rate and treatment failure, a random effects model meta-analysis conducted of the studies showed no difference in rate between cefepime and comparator antibiotics with an overall risk ratio of 0.98 (95% CI: 0.92–1.05) and 1.04 (95% CI: 0.91–1.19), respectively. Adverse events were not statistically assessed given widespread heterogeneity. Overall, the studies had unclear risk of bias and were limited by high heterogeneity and methodological flaws.

**Conclusion:**

The efficacy and safety of cefepime in pediatric patients remain unclear despite the inclusion of newer trials since the last index systematic review conducted a decade ago.

## Background

Cefepime is a fourth-generation cephalosporin antibiotic used most commonly in the clinical setting for its beta lactamase resistance and activity against *Pseudomonas aeruginosa*. In 1999, the U.S. Food and Drug Administration (FDA) approved cefepime for use in children greater than 2 years. Its main uses included the treatment of moderate-to-severe pneumonia, urinary tract infection (UTI), skin and soft tissue infection, and complicated intra-abdominal infections. It has also been used for the empiric treatment of febrile neutropenic patients (1).

Two prior systematic reviews by Paul et al. in 2006 and Yahav et al. in 2007 investigated the use of cefepime in comparison to other beta lactam agents or cephalosporins after several randomized clinical trials suggested that treatment with cefepime was associated with increased odds of mortality (2, 3). Following the findings of these reviews, the FDA called for further investigation (4). Opposing results in 2009 demonstrated that cefepime was indeed safe for use in pediatric patients (5). These conflicting results pointed toward the need for further examination. Another systematic review and meta-analysis was performed by Adderson et al. in 2010, aiming to evaluate subgroups of pediatric patients by etiology and compare use of cefepime versus conventionally used antibiotic regimens (6). Adderson et al. included sixteen clinical trials but found that the evaluation of the efficacy and safety of cefepime was limited by the small number and poor quality of trials. However, they concluded that pediatric patients who are treated with cefepime are not at increased risk of adverse outcomes. This conclusion is limited to patients older than 2 years. The FDA still recommends that clinicians use cefepime with caution, and adequate safety information remains scarce for infants age 0–2 years. As drug resistance becomes more problematic, demanding use of increasingly potent antibiotics to empirically cover acutely ill patients, cefepime use has become ubiquitous in hospital settings (7). This increased use and questionable safety profile calls for re-evaluation of the available literature. Therefore, the main objective of this review is to compare all-cause mortality following use of cefepime and overall efficacy and safety of cefepime compared to other clinically indicated antibiotics among children.

## Methods

We structured the review following the PICOTS (Population, Intervention, Comparator, Outcome, Timing, Setting) framework. Our study population (P) included pediatric patients aged 0–19 years with fever, elevated white blood cell count, or other clinical signs of infection. The evaluated intervention (I) was intravenous (IV) or intramuscular (IM) cefepime, used in standard pediatric doses (50 mg/kg every 12 h to 2 g every 8 h, with a maximum dose of 6 g per day) and duration of treatment (10–14 days). Comparators (C) included: IV or IM beta lactams other than cefepime, carbapenem, aminoglycoside, or a “clinically indicated” antibiotic treatment. Studies comparing cefepime versus placebo or cefepime versus no treatment were also analyzed. The primary outcome (O) of this study was 30-day all-cause mortality defined as being dead or alive 30 days after the onset of treatment with cefepime. Mortality at the end of the study period was used as reported when the 30-day all-cause mortality was not clearly specified.

This dichotomous outcome was an aggregated number of deaths per trial arm. Additional secondary outcomes of interest were as follows: (1) success rate, defined as resolution of fever with improvement of clinical symptoms without signs of infection, (2) treatment failure, defined as clinical or microbiological evidence of persistent infection after full course of treatment, or the addition of a second agent covering same spectrum, and (3) incidence of adverse events including antibiotic-associated morbidity, defined as irreversible antibiotic-related adverse effect such as hepatotoxicity, nephrotoxicity, or neurotoxicity. Timing (T): outcomes were measured for up to 30 days following completion of treatment, or until the end of the index hospital admission, if lesser than 30 days. The follow-up period was as defined by individual studies or ended at the conclusion of the study if not clearly specified. Setting (S): the study was limited to inpatient hospital administration of antibiotics. This study was registered in the International Prospective Register of Systematic Reviews called PROSPERO^1^ (registration # CRD42016036515).

### Study Eligibility

#### Types of Studies

For this review, included studies were restricted to randomized controlled trials comparing cefepime to other clinically indicated antibiotics. Open-label studies were permitted because of the general scarcity of research conducted in pediatric trials in the area of interest. All non-randomized and observational studies were excluded from the review.

#### Study Population

Study participants were patients aged 0–19 years in the inpatient setting who had clinical signs of infection such as fever, elevated white blood cell count, elevated heart rate or respiratory rate, low blood pressure, suspicion of infection (patients undergoing chemotherapy at high risk for infection who have minimal symptoms), or proven infection (culture data documented for infection). There were no restrictions on type of infection (urinary, pulmonary, skin, soft tissue, etc.) so long as cefepime was deemed clinically appropriate by study personnel. We included patients with “fever of unknown origin” being empirically treated prior to isolation of an infectious source. No restrictions were placed on gender, ethnicity, co-morbidity, or number of participants. For age, we restricted our participants to those less than 19 years. For studies that included participants crossing over our maximum age of inclusion, we included the study if we were able to extract separate data for our population of interest.

#### Interventions

We included studies comparing standard pediatric doses of cefepime to controls. Controls included any other IV or IM antibiotics found to be clinically indicated by the authors based on the underlying disease process. We also included studies comparing cefepime to placebo or to non-treatment, which is most appropriate in patients being treated empirically for “fever of unknown origin.” Intervention was restricted to IV/IM routes of administration, excluding oral and other forms of administration (e.g., peritoneal). In terms of dosing, we included studies using any standard pediatric doses and duration of treatment (50 mg per kg every 12 h to 2 g every 8 h, maximum dose is 6 g per day; duration based on specific disease course approximately 10–14 days).

#### Outcome Definition

The primary outcome was 30-day all-cause mortality, a dichotomous variable defined as being dead or alive 30 days after the onset of treatment. Mortality at the end of the study period was used as reported when the 30-day all-cause mortality was not clearly specified. Our secondary outcomes included: (1) success rate, defined as resolution of fever with improvement of clinical symptoms. This outcome was subdivided into two categories: overall success rate (total number of cases that improved, regardless of whether a different antibiotic was added) and success rate without modification (cases who improved prior to adding another antibiotic) which was reported by some studies. (2) Treatment failure, defined as clinical or microbiological evidence of persistent infection after full course of treatment, or the addition of a second agent covering same spectrum. (3) Incidence of adverse events was the third (listed below) including antibiotic-associated morbidity, defined as irreversible antibiotic-related adverse effects such as hepatotoxicity, nephrotoxicity, or neurotoxicity.

### Search Strategy

Our search included MEDLINE, EMBASE, CENTRAL, LILACS, and clinicaltrials.gov databases. No restrictions were placed on the years covered by each database. Our search strategies are included in the attached Supplementary Material. We searched Micromedex and EMBASE for synonyms of cefepime in any language, including brand name products. Our search strategies aimed to include published studies as well as potentially relevant unpublished gray literature. Our searches had no restrictions with regard to language, publication year, or population age. We searched the National Institute of Health’s clinical trial registry^2^ to identify potentially relevant unpublished studies. The date of search was documented for all electronic searches. The final search of all databases was conducted through February 8, 2016.

### Data Collection and Analysis

#### Data Extraction and Management

To reduce bias and maintain the validity of the review, each title and abstract was screened independently by two members of the team (double screened). All authors participated in this process and articles for full text review were selected by consensus between each member pair or by inviting another member of the team to solve discrepancies (8). Covidence tool was used in full text screening to review studies included in the systematic review with a double screening protocol (9). Discrepancies were resolved as specified above. For details of the extracted data form each study, see Supplementary Material. Double data extraction by two independent team members was used for data extraction to ensure accuracy. Differences were also jointly resolved as needed.

#### Data Analysis

Based on the binary nature of our primary and secondary outcomes, we employed relative risk as our outcome measure. From our primary review of data in this field, we concluded that studies were heterogeneous in intervention, population, and other factors. For this reason, a random effects model was most appropriate to capture effect size and account for the variance between and within models. The Systematic Review and Data Repository tool was used for managing data throughout the course of this systematic review and meta-analysis (10). In addition to keeping records of data, this tool was used for data extraction. For quality assurance, two members of the team independently extracted data from each study and entered it into the form created. We performed data comparisons by hand between the two team members extracting from the same study and ensured that the data entered by both team members matched. Discrepancies were accordingly resolved either by the two team members revisiting the study or by a third member of the team as a mediator (11). The extracted data was then loaded into RevMan 5.2 for analysis (12). A random effect model meta-analysis was conducted to estimate the risk ratio of the outcomes.

#### Assessment of Risk of Bias in Included Studies

Risk of bias assessment was performed independently by two reviewers per study. Reviewers of each study were not blinded to the authors or journals of publication. The studies were evaluated for risk of bias using criteria adapted from the Cochrane Risk of Bias Assessment Tool and the Higgins et al., 2008 Cochrane handbook reference. Discrepancies in assignment of the risk of bias were discussed between the two assigned reviewers, or resolved with a third team member if consensus was not reached. Criteria for definition of high, low, or unclear risk of bias are included in Supplementary Material.

#### Assessment and Investigation of Heterogeneity

We examined clinical and methodological heterogeneity by qualitatively assessing differences across studies. Methodological heterogeneity was attributable scarcity of studies about this topic in the pediatric population. Given the relatively low number of pediatric trials compared, we included open-label studies. Nonetheless, we aimed to analyze open-label trials separately, considering the high risk of performance bias. Clinical heterogeneity was attributable to comparing characteristics among studies that could contribute to different outcomes. For example, clinical outcomes could be heterogeneous when comparing population subgroups among pediatric patients (female versus male, chemotherapy versus no chemotherapy), routes of administration (IV versus IM), dosages of cefepime, or unique combinations of cefepime tested in intervention arms with different antibiotics. Variations in the definitions of primary and secondary outcomes, including the timing of outcomes, were assessed as well.

Statistical heterogeneity was assessed by *I*^2^ statistic calculation, and we set our cutoff at 60% for acceptability in permitting our analysis. The *I*^2^ statistic as a numerical calculation allowed us to better assess heterogeneity as an estimate of variance on a relative scale. An *I*^2^ statistic of 60% is considered between moderate and high heterogeneity as suggested by Cochrane. Given these guidelines, we decided *a priori* that we would not conduct a meta-analysis if our *I*^2^ statistic exceeded this value. Study results were illustrated through a collective forest plot with confidence intervals included for comparison.

#### Post-Protocol Changes to Methods

The protocol was submitted to the research committee at Johns Hopkins Bloomberg School of Public Health. There were several critical changes to methods that were made after formation of our protocol. Most notably, we had originally defined our secondary outcomes as (1) treatment failure, (2) antibiotic-associated morbidity, and (3) incidence of adverse events. However, when we subsequently re-visited our secondary outcomes, we changed our outcomes to be (1) success rate, (2) treatment failure, and (3) adverse events—as described in sections above. This was, in part, due to the need to include success rate as an outcome measuring our objective for efficacy, particularly since a majority of the papers presented these data through their results. Moreover, in studies where success rate without modification was provided as an outcome in addition to overall success rate, it was decided that success rate without modification would be examined to better correspond to the true effect of cefepime. Furthermore, our original protocol had not specified implications of participants in study arms being characterized and/or randomized by episodes of febrile neutropenia instead of by number of participants. We made an addendum to specify this under the participants and inclusion criteria. Additionally, follow-up time was listed in our original protocol as 30 days or until no longer hospitalized, but this was changed to a flexible window given that many studies did not specify a follow-up time. Thus, this ambiguity was accepted regardless of whether follow-up was specifically defined or not by the studies.

## Results

### Description of Studies

**Figure 1:**
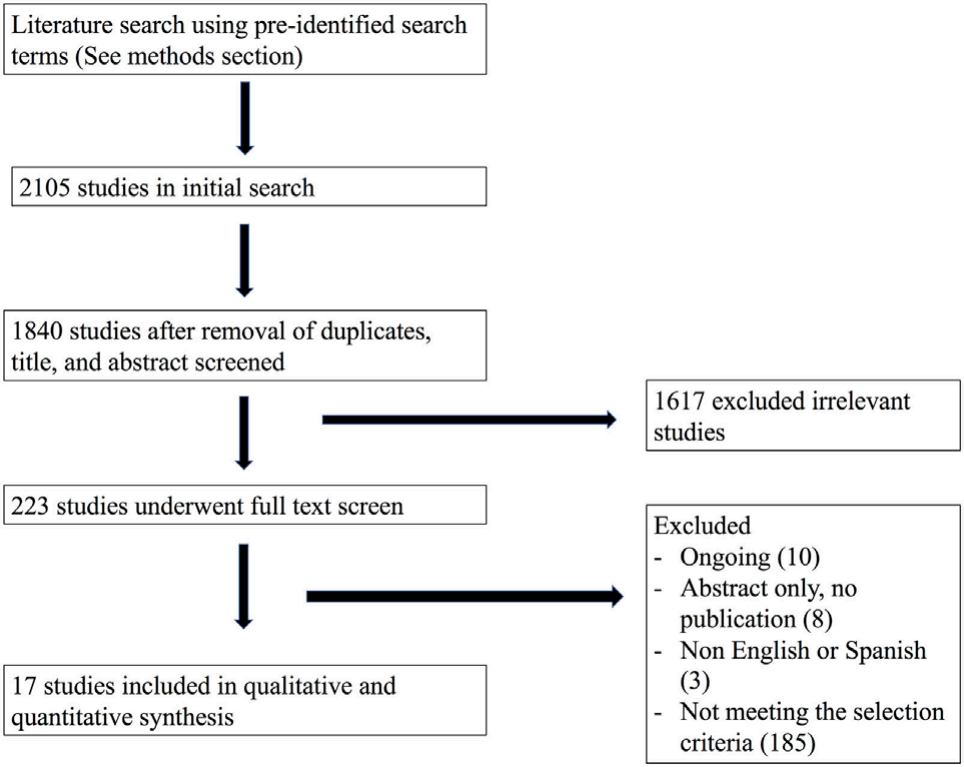
Study selection flow chart.

Our initial search from the MEDLINE, EMBASE, CENTRAL, LILACS, and Clinicaltrials.gov databases in February, 2016, yielded a total of 1,840 titles and abstracts for initial screening after duplicate removal. Subsequently, after initial abstract and title screen, 223 articles were selected for full text review. Finally, using our pre-defined PICOTS criteria, 17 studies were included in the final analysis. Our study search and selection process are summarized in Figure 1.

There were a total of 1,285 subjects included in the selected studies, with 624 participants in the cefepime arm and 661 in the comparative antibiotic arm. Participants’ indication for the use of cefepime varied among studies. The most common reason for cefepime use was febrile neutropenia (12 studies). The remaining studies, listed pneumonia, complicated UTI, bacterial meningitis, and/or intra-abdominal infection as indications for cefepime use. Age ranges encompassed infancy to adolescence (0–19 years) except for Shahid’s study, which only included infants under 1 year (13). Table 1 shows a summary and characteristics of the included studies.

**Table 1:** Evidence and characteristics of included studies.

Reference	Type of RCT	Participants	Comparator	Relevant outcomes	Highlighted results (95% CI)	Favors
Mustafa et al. (15)	Single center, open label	2 months–18 years old children with febrile neutropenia	Ceftazidime	- Success rate	1.11 (0.56, 2.21)	Either
				- Treatment failure	0.85 (0.37, 1.97)	
				- Mortality	None	

Aamir et al. (21)	Single center	40 children, <18 years with febrile neutropenia	Piperacillin/tazobactam	- Success rate	1.33 (0.30, 5.93)	Either
				- Treatment failure	0.80 (0.25, 2.55)	
				- Mortality	2.0 (0.41, 9.71)	

Cannavino et al. (25)	Multi-center	3 months–18 years old children with intra-abdominal infection, complicated UTI, pneumonia	Doripenem	- Cure	N/A	Either

Shahid (13)	Single center, pilot	30 infants, <1 year old with ventilator-associated pneumonia, excluding preemies	Ceftazidime	- Success rate (eradication)	4.0 (0.88, 18.26)	Either
				- Failure	1.00 (0.24, 4.18)	
				- Mortality	0.61 (0.06, 5.86)	

Pereira et al. (16)	Single center, open label	57 children, 0- to 21-year-old, 130 episodes febrile neutropenia related to stage III and IV lymphoma	Ceftriaxone plus amikacin	- Success rate	1.06 (0.36, 3.13)	Either
				- Success with modification	N/A	
				- Treatment failure	1.06 (0.54, 2.10)	
				- Mortality	0.97 (0.06, 14.70)	

Kebudi et al. (20)	Single center	31 children with solid tumors, 40 episodes febrile neutropenia	Ceftazidime	- Success rate	0.80 (0.29, 2.23)	Either
				- Success without modification	N/A	
				- Success with modification	N/A	
				- Mortality	None	

Kutluk et al. (29)	Single center, single blind	30 children, <16 years old with lymphoma and solid tumors, 49 episodes febrile neutropenia	Meropenem	- Success rate	0.30 (0.07, 1.32)	Either
				- Mortality	0.96 (0.06, 14.50)	

Kebudi et al. (23)	Single center	31 children, 40 episodes febrile neutropenia	Piperacillin/tazobactam	- Success with modification	1.11 (0.26, 4.72)	Either
				- Success without modification	N/A	
				- Mortality	0.96 (0.06, 14.50)	

Chuang et al. (14)	Single center, open label	58 children, 2 months–15 years old, with febrile neutropenia	Ceftazidime	- Success rate	0.91 (0.38, 2.17)	Either
				- Treatment failure	1.04 (0.70, 1.54)	
				- Mortality	1.47 (0.2, 8.38)	

Corapcioglu et al. (22)	Single center	50 children, ≤18 years old with febrile neutropenia	Piperacillin/tazobactam	- Success without modification	0.73 (0.24, 2.21)	Either
				- Failure (persistent fever)	None	
				- Mortality	None	

Sano et al. (24)	Single center	53 children, <22 years old, with malignancy treated with chemotherapy, having febrile neutropenia	Piperacillin/tazobactam	- Success rate	0.88 (0.51, 1.53)	Either
				- Failure (new infection)	None	
				- Mortality	0.31 (0.01, 7.58)	

Sarashina et al. (28)	Multi-center	64 children, ≤21 years with 223 episodes of febrile neutropenia	Cefozopran	- Success rate	0.72 (0.42, 1.24)	Either
				- Failure (new infection)	None	
				- Mortality	None	

Schaad et al. (17)	Multi-center, third party blinded, open label	300 children, 1 month–12 years old with pyelonephritis	Ceftazidime	- Success rate	2.46 (0.47, 12.92)	Either
				- Failure	0.70 (0.12, 4.09)	

Uygun et al. (19)	Single center, open label	69 children, ≤19 years, with 127 episodes of febrile neutropenia	Piperacillin/tazobactam	- Success rate	1.94 (0.19, 21.91)	Either
				- Treatment failure	0.52 (0.05, 5.64)	
				- Mortality	0.53 (0.05, 5.64)	

Oguz et al. (18)	Single center, open label	48 children with 65 episodes febrile neutropenia, solid tumors	Meropenem	- Success rate	1.24 (0.45, 3.41)	Either
				- Treatment failure	0.87 (0.46, 1.65)	
				- Mortality	None	

Saez-Llorens et al. (27)	Single center	90 children 2 months-15 years old, with bacterial meningitis	Cefotaxime	- Success rate	N/A	Either
				- Mortality	0.55 (0.11, 2.83)	

Janssen Research & Development, LLC (26)	Multi-center, double blind	40 children, 3 months–18 years old with complicated UTI	Doripenem	- Success rate	0.50 (0.12, 2.14)	Either

*RCT, randomized clinical trials; N/A, not available*.

There were 4 multi-center studies and 13 single center studies across Asia, Europe, South America, and North America. The country of Turkey was particularly well represented as the site of several studies. There were six author-disclosed open-labeled studies (14–19).

### Interventions

Cefepime was compared to several other antibiotics, including ceftazidime (13–15, 17, 20), piperacillin/tazobactam (19, 21–24), doripenem (25, 26), meropenem, cefotaxime (27), cefozopran (28), and ceftriaxone-amikacin (16). Nearly all of the studies had IV as the route of administration with the exception of Aamir et al., which permitted both IV/IM (21). The dosage, frequency, and duration of both intervention and comparator antibiotics varied throughout the studies. The length of the intervention duration was often between 7 and 14 days, and seven studies did not specify the duration of antibiotic therapy (13, 16, 20, 22, 25, 26, 28).

### Outcomes

Most studies provided mortality figures or specifically stated that there were no deaths during the course of the study. There was no mention of our primary outcome of mortality in three studies (17, 25, 26). In regard to our secondary outcomes, success rate was included in nearly all the studies either as the overall success rate or success rate without modification (improvement prior to addition of another antibiotic). Following our protocol, success rate without modification was preferentially used if provided to best approximate the effect of cefepime. Treatment failure was reported in all but six studies (20, 23, 25–27, 29). Regarding adverse events, only 9 of the 17 studies recorded this outcome. The most commonly reported adverse events were diarrhea and rash in six and five studies, respectively. Additional adverse events included rash, diarrhea, nausea, vomiting, pruritus, headache, eosinophilia, candidal mucosal superinfection, elevated liver enzymes, renal impairment, epistaxis, cough, abdominal pain, pseudomembraneous colitis, UTI, and hypokalemia.

### Risk of Bias Assessment

**Figure 2:**
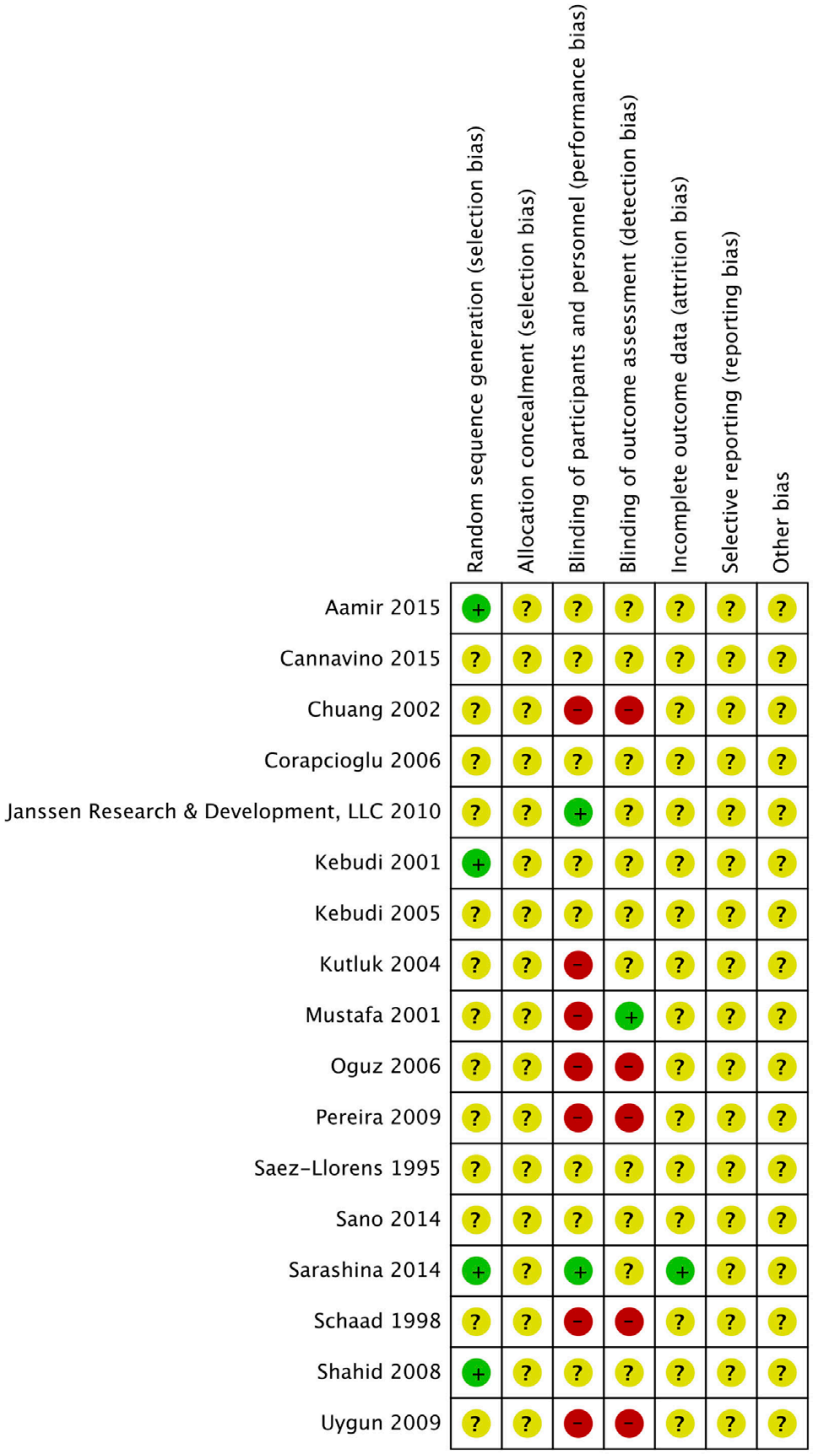
Risk of bias summary of the included studies.

**Figure 3:**
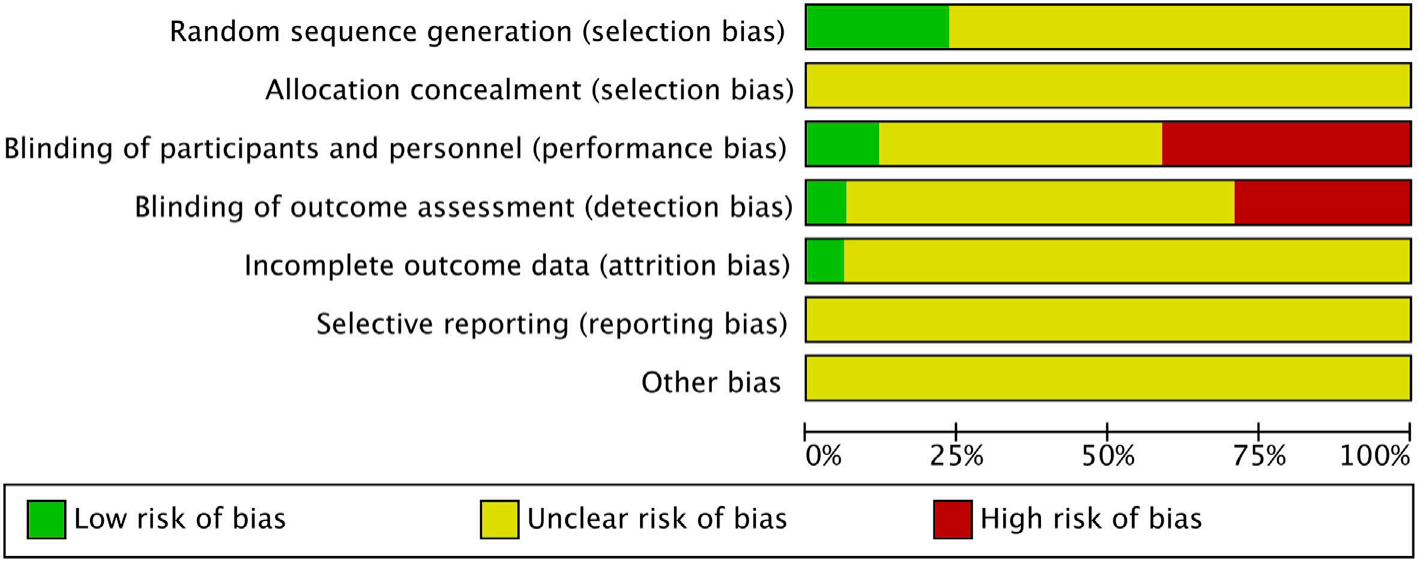
Risk of bias graph of the included studies.

Risk of bias was assessed in all 17 studies using the Cochrane adapted tool (30). Most studies included were classified as unclear risk of bias due to unreported details of risk of bias assessment per criteria and designated levels. High risk of bias was reported in seven studies due to lack of blinding of participants and personnel (performance bias) (14–19, 29) and reported in five studies due to lack of blinding of outcome assessment (detection bias) (14, 16–19). Sarashina et al. is the only study with low risk of bias in three categories of risk of bias assessment (28) (Figures 2 and 3). A summary of our risk of bias assessment is included in Figures 2 and 3.

#### Allocation

In four studies, random sequence generation was used in randomization (13, 20, 21, 28). Aamir et al. made use of a computer generated random number for randomization (21). The study by Kebudi 2001 only reported that “randomization was made using random numbers” (20). Sarashina et al. also expressed allocation concealment as part of the methods supporting a low risk of bias for the designation of this criteria (28). Sarashina et al. reported that randomization was done in an “evaluator-blind fashion, with assignment of unique numbers to participants on the appropriate stratified list” (28). Shahid et al. made use of a computer generated random table (13) Allocation was reported to be concealed in envelopes and was unknown to investigators.

#### Blinding

Six studies were identified as open-label studies with no blinding among participants or personnel for the study arms (14–19). None of them were masked for outcome assessment except Mustafa et al., which clearly stated masking of outcome assessment (15). These studies were flagged for high risk of bias. Kutluk et al. was claimed to be a single-masked study by the authors, implying that patients were masked to treatment, but this was not clearly stated in the paper, indicating a high risk of bias (29). Two studies described masking of outcome assessors granting a low risk of bias for those categories, respectively, for the studies (26, 28). The remaining studies had unclear masking.

#### Incomplete Outcome Data

Bias related to incomplete data for attrition was unclear upon assessment of the included studies. There were no explicit statements in any of the studies regarding how authors handled missing data.

#### Selective Reporting

Selective reporting was unclear in assessment of the included studies. However, one marker of selective reporting is the complete lack of reporting of adverse events in seven studies. It is highly doubtful that there were no adverse events suffered by any of the participants in these studies; however, there was no clear documentation of serious or even mild adverse events. Moreover, there was a lack of information for clearly defined follow-up time across studies.

#### Funding

Most studies mentioned have no disclosures to report and no major conflicts of interest. Cannavino et al. disclosed having a pharmacologist and statistician with relevant financial ties, and Saez-Llorens et al. disclosed funding support from pharmaceutical company Bristol-Myers Squibb (25, 27).

### Quantitative Synthesis

#### Primary Outcome

**Figure 4:**
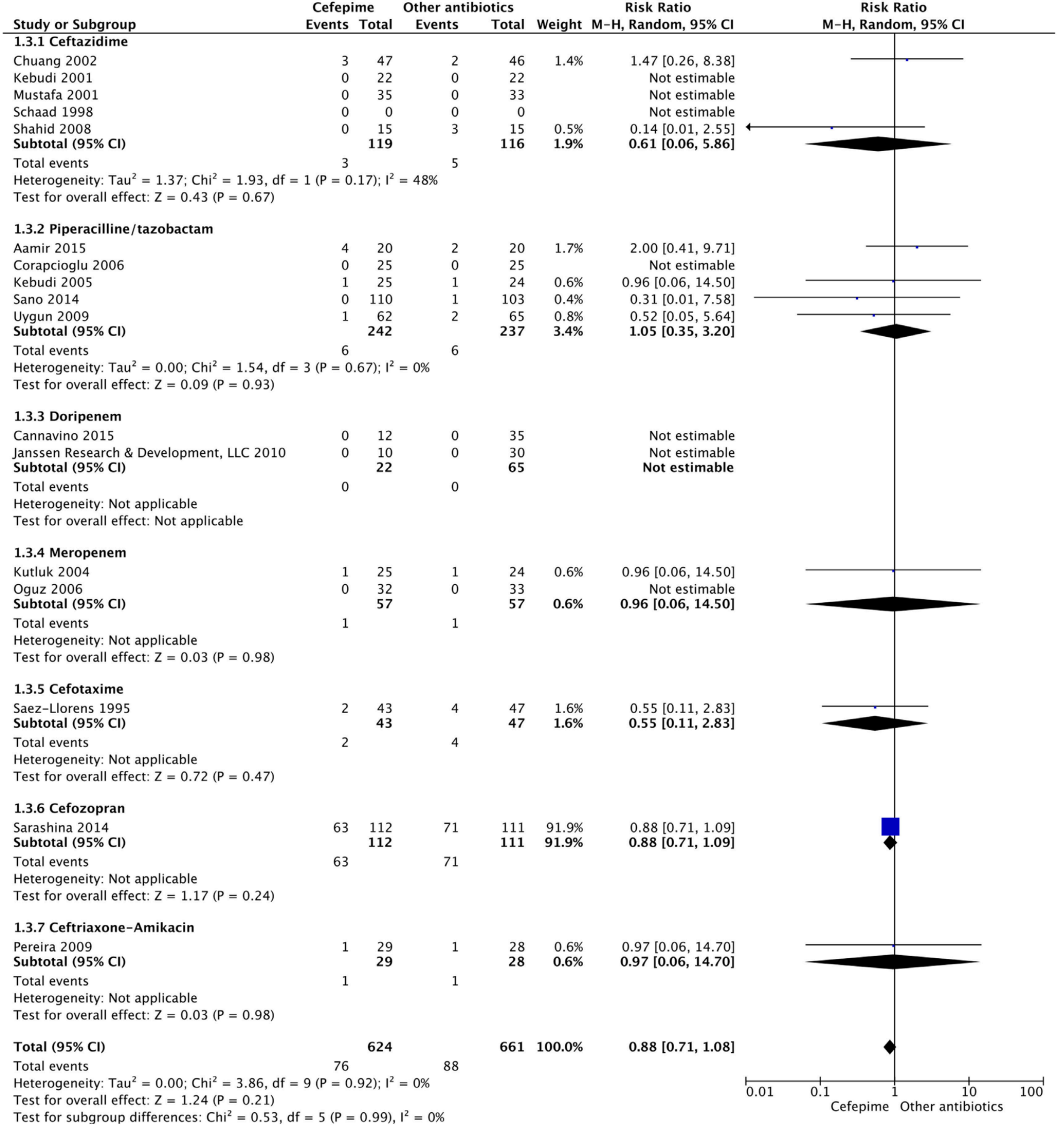
All-cause mortality for cefepime versus comparator.

For the primary outcome of all-cause mortality, events were compiled for all the studies that had mortality data. A random effects model meta-analysis conducted of the studies gave an overall mortality risk ratio of cefepime to comparator antibiotics of 0.88 (95% CI: 0.71–1.08) with *I*^2^ = 0%, τ^2^ = 0.00, *p*-value = 0.92 for Chi^2^ test (Figure 4). This suggests no statistically significant difference between cefepime and comparator antibiotics, on the outcome of mortality. A sensitivity analysis was conducted after removing all studies deemed to have a high risk of bias (14–19, 29). Following this, the overall mortality risk ratio of cefepime to comparator antibiotics was adjusted to 0.77 (95% CI: 0.30–1.97) with *I*^2^ = 0%, τ^2^ = 0.00, *p*-value = 0.51 for Chi^2^ test.

#### Secondary Outcomes

**Figure 5:**
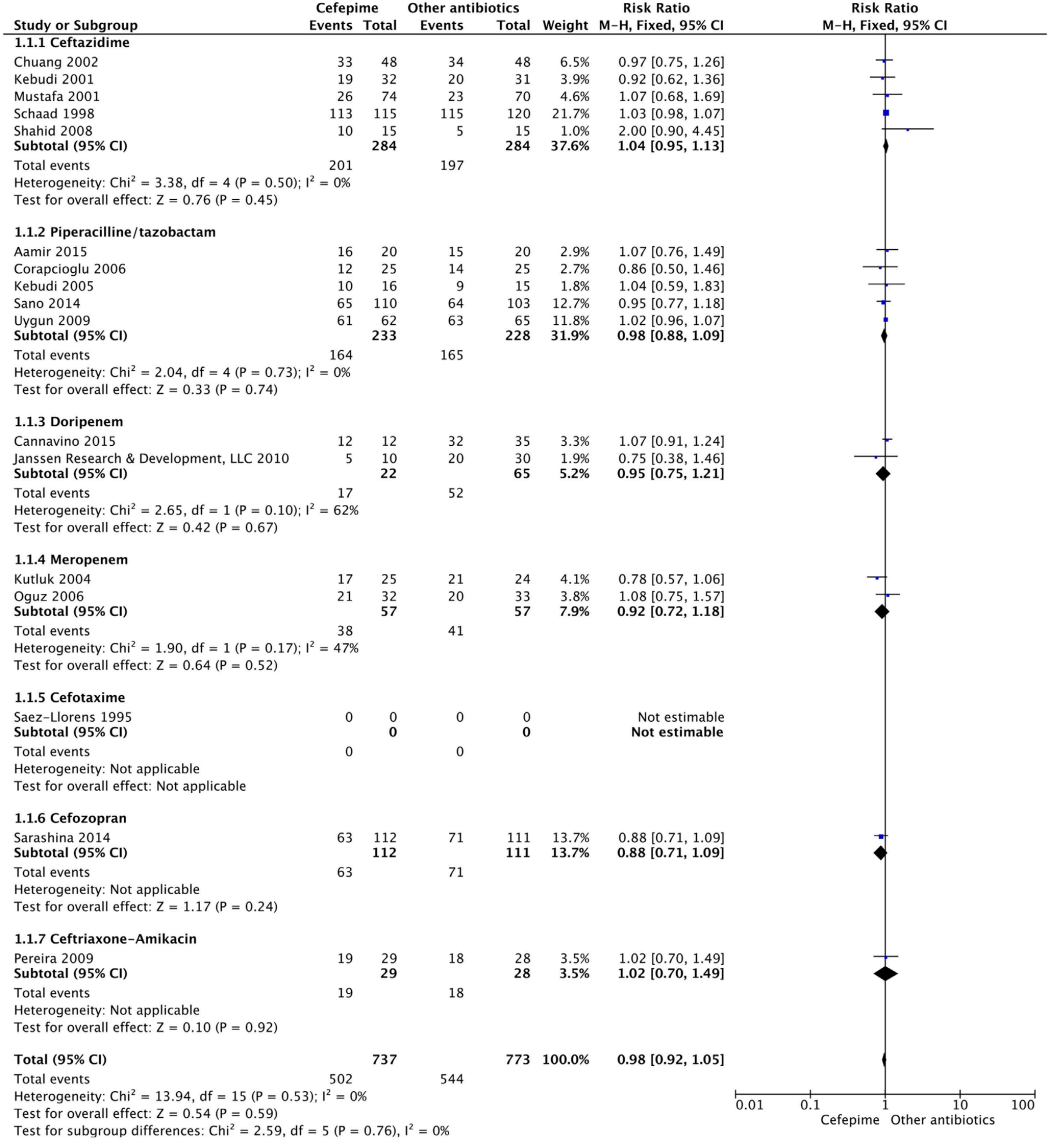
Success rate for cefepime versus comparator.

**Figure 6:**
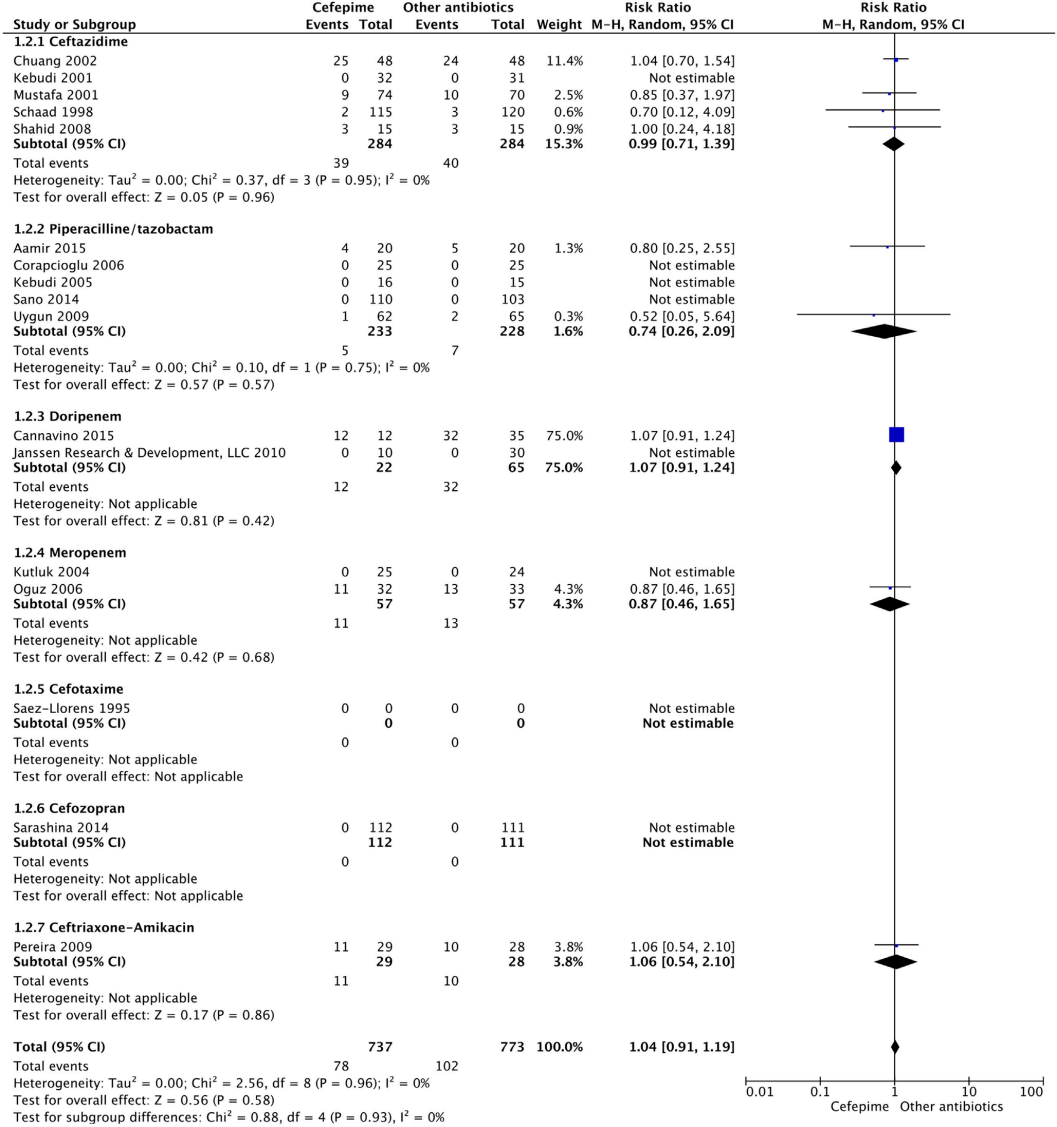
Treatment failure for cefepime versus comparator.

For the secondary outcome of success rate, a random effects model meta-analysis conducted of the studies gave an overall risk ratio of cefepime to comparator antibiotics of 0.98 (95% CI: 0.92–1.05) with *I*^2^ = 0%, τ^2^ = 0.00, *p*-value = 0.53 for Chi^2^ test (Figure 5). Removing all high risk of bias studies in a sensitivity analysis, we found the overall risk ratio to be 0.99 (95% CI: 0.90–1.09) with a *I*^2^ = 0%, τ^2^ = 0.00, *p*-value = 0.53 for Chi^2^ test. Fewer studies reported treatment failure, as described in a previous section, but the random effects model meta-analysis conducted of studies provided an overall risk ratio of cefepime to comparator antibiotics of 1.04 (95% CI: 0.91–1.19) with *I*^2^ = 0%, τ^2^ = 0.00, *p*-value = 0.96 for Chi^2^ test (Figure 6). The sensitivity analysis subsequently conducted removing all high risk of bias studies gave an overall risk ratio of 0.87 (95% CI: 0.35–2.15) with *I*^2^ = 0%, τ^2^ = 0.00, *p*-value = 0.81. Adverse events were not statistically assessed given widespread heterogeneity. However, the total number of adverse events per study is listed in Table 2.

**Table 2:** Number of total adverse events reported from cefepime and comparator arms.

Reference	Total number of participants	Cefepime,*N*	Comparator,*N*
Aamir et al. (21)	40	6	5
Cannavino et al. (25)	88	5	12
Corapcioglu et al. (22)	50	2	0
Chuang et al. (14)	95	1	0
Mustafa et al. (15)	104	33	37
Pereira et al. (16)	57	4	9
Saez-Llorens et al. (27)	90	18	24
Schaad et al. (17)	299	9	9
Uygun et al. (19)	69	13	13
Janssen Research & Development, LLC (26)	41	12	7
Shahid (13)	30	Non-reported	Non-reported
Sano et al. (24)	53	Non-reported	Non-reported
Sarashina et al. (28)	64	Non-reported	Non-reported
Oguz et al. (18)	37	Non-reported	Non-reported
Kebudi et al. (20)	44	Non-reported	Non-reported
Kebudi et al. (23)	31	Non-reported	Non-reported
Kutluk et al. (29)	30	Non-reported	Non-reported

*N, number*.

## Discussion

To our knowledge, this study is the most updated systematic review of studies investigating the safety of cefepime use in pediatric patients. Our results failed to demonstrate statistically significant differences in mortality or adverse events among children treated with cefepime. Additionally, no significant differences were found in treatment success and treatment failure in patients treated with cefepime when compared with other antibiotics used for similar indications in the studies analyzed.

However, it is important to take into consideration the general nature of the studies included and implications of their design. Building upon what was detailed in previous results sections, it is necessary to stress the high level of methodological flaws and heterogeneity that existed among the included publications. Specifically, when presenting outcomes, several of the studies used number of febrile episodes as a central denominator, instead of participants, for randomization. This has severe implications for the results because there was not specification whether such trials had participants who received both cefepime as well as another antibiotic for different febrile episodes. Nearly resembling crossover studies, these design flaws cause major reservations in the interpretability of such studies. Moreover, despite most trials specifying interval duration of treatment, nearly none of the studies went through to clearly define the length of follow-up time for outcomes. We relaxed our restrictive protocol to accommodate the reality of available studies. In regard to secondary outcomes, treatment failure and adverse events outcome were assessed in only 11 and 9 studies out of 17, respectively.

Definitions for outcomes had some variation between studies. Namely, success rate was presented both as an overall rate in some studies, as a rate without modification in others, and potentially both in some publications. One potential problem is that these outcomes were measured differently across studies so may not be completely comparable. However, our results raise the question of whether cefepime should be used before other antibiotics with similar efficacy when equivalent choices are available with no concerns regarding safety. Our modified protocol allowed us to navigate this barrier to our best ability, but the level of heterogeneity and methodological flaws in the trials are important to understand while contemplating the results.

Compared to the 2007 index review by Yahav et al., a total of 57 trials of adult and pediatric population with 5 trials of only pediatric population were included. The all-cause mortality was higher with cefepime compared to other β-lactams antibiotics with a risk ratio of 1.26 (95% CI: 1.08–1.49). After sensitivity analysis, the risk ratio was increased to 1.52 (95% CI: 1.20–1.92) and 1.36 (95% CI: 1.09–1.70) for trials reporting adequate allocation-sequence generation and allocation concealment, subsequently. There are high-quality methodological trials among the adult population reported in that review. For example, in the adult population trials, adequate allocation concealment and random sequence generation were reported in 30 trials, whereas in the pediatric population trials that we reviewed, no trials had adequate allocation concealment and only four had random sequence generation. Moreover, masking of outcome assessors was conducted in eight adult trials compared to two trials in our review. Although these findings might raise a concern for increased risk of mortality with cefepime use in high-quality methodological trials among adult population, an up-to-date systematic review including the adult and pediatric population is warranted to evaluate all new trials since 2007. The FDA report in 2009 included 88 trials among both the adult and pediatric population, and showed an increase in mortality that was not statistically significant following cefepime use in both the adult and pediatric populations. There was not a sensitivity analysis performed including only the trials deemed to have high-quality methodology. Conducting a sensitivity analysis to assess for all-cause mortality in only high-quality trials is essentially impossible in our review given the low-quality methods for most trials, as stated in Section “Results.”

Compared to the 2010 index review by Adderson et al., there are many similarities to be noted. First, both our review and the index review seemed to draw the same general conclusions with regard to efficacy and safety of cefepime. Their risk ratio for all-cause mortality was given as 1.11 (95% CI: 0.59–2.10) with *I*^2^ = 0%, *p*-value = 0.66, and their treatment failure risk ratio was 0.93 (95% CI: 0.82–1.04) with *I*^2^ = 0%, *p*-value = 0.91. These values were like our figures and not statistically significant. Compared with our review, the methods from Adderson et al. differed slightly in breaking down analysis by subgroup of disease (febrile neutropenia versus others) and focused on the outcomes of mortality and treatment failure only. Their review also included a table of reported adverse events per arm by study but, as we similarly concluded, further analysis was challenging due to the degree of heterogeneity of reported outcomes. Notably, our review was able to include several studies not previously included in the index review (16, 19, 21, 23–26, 28).

Our review has several limitations that must be acknowledged. Besides the general poor quality of studies analyzed for this review as already discussed, we had limited capacity to rigorously further explore information not readily extracted. Despite attempts to contact study authors to clarify details and secure further data not explicitly stated in published manuscripts, we were not successful in gaining more information. Given our restrictions of the authors to English and Spanish languages, we were limited in ability to explore three potentially relevant studies that were in other languages—most notably Chinese. However, these studies did not necessarily pass full text review, and it is unknown whether they would have met inclusion criteria. Although our main outcome was reported consistently in most studies and it is was a robust variable, it was not reported in three studies possibly biasing our results. The heterogeneity of definitions for secondary outcomes affected our analysis. Success rate was defined as an overall success rate in some studies, as a rate without modification in others, and potentially both in some publications. Thus, findings about secondary outcomes indicating that they are not different should be taken with extreme precaution. The evaluation of the intervention (cefepime) is also problematic as several doses and duration of treatment were used. As resistance to third generation cephalosporins has increased and as nosocomial infections have become a more threatening trend for inpatients, it is imperative that we consider the role of cefepime use in the in-hospital setting and are mindful of the safety trend over time. Nonetheless, our study is a valuable update to the topic and provides a comprehensive systematic review of the literature and some valuable insights to consider when considering cefepime use in pediatric patients. Moreover, it provides an incentive for further exploration of this important clinical problem in the treatment of severe pediatric infectious events.

## Conclusion

Following a rigorous systematic review, we cannot propose changes or strong clinical recommendations in contrast to current practice in the use of cefepime in the pediatric population. Cefepime may be cautiously used in clinical practice as indicated, as there does not seem to be any statistically significant difference for the antibiotic compared to a variety of others in the rates of mortality, treatment success, and treatment failure. However, the poor quality of studies available for review and their limitations suggest that current evidence may not necessarily describe the truth. Despite several new trials since the last systematic review, our findings do not reflect different results. Further prospective research and higher quality trials are needed to better capture the clinical consequences of cefepime use in pediatric patients. Our recommendation is for investigators to adhere to the strongest trials criteria to avoid bias while explicitly detailing this methodology in their published manuscript or to include an accompanying protocol. Going forward, an updated systematic review and meta-analysis will be warranted once results of ongoing trials and more robust studies in children accrue, to reevaluate the efficacy and safety of cefepime in the pediatric population.

## Author Contributions

SJ, BR, SD, OA, and MG conceptualized and designed the study, and designed the data collection instruments. SJ, BR, SD, OA and MG performed the data extraction and coordinated data collection and analyses. SJ drafted the initial manuscript. BR, SD, OA, and MG reviewed and critically revised the drafts of the manuscript and approved the final manuscript as submitted.

## Conflict of Interest Statement

The authors declare that the research was conducted in the absence of any commercial or financial relationships that could be construed as a potential conflict of interest.

## Footnotes

^1^www.crd.york.ac.uk/prospero.

^2^www.clinicaltrials.gov.
