# Supplementary material for: Cefepime Efficacy and Safety in Children: A Systematic Review and Meta-analysis
Source: Front Pediatr. 2018 Mar 6;6:46. doi: 10.3389/fped.2018.00046 (PMC5845692; doi:10.3389/fped.2018.00046)
Supplement: Supplementary file 1 [file table_1.docx]

**Cefepime Efficacy and Safety in Children: A Systematic Review and Meta-Analysis**

Saber Jan ^1,2^, Braveen Ragunanthan ^3^, Sandra R. DiBrito ^4^, Omolabake Alabi ^5^, Maria Gutierrez ^6 *^

**Supplementary materials: Table**

**Table 1: Tool used to assess risk of bias adapted from Cochrane**

| Table 2. Risk of bias evaluation tool, adapted from Cochrane Risk of Bias Tool. | | | | |
| --- | --- | --- | --- | --- |
| Type of Bias | Criteria | High Risk | Low Risk | Unclear |
| Selection | Random sequence generation | The authors describe a non-random method of sequence generation | The authors describe a method of randomized sequence generation (i.e. computer generated, tossed coin, etc.) | There is no enough information to distinguish “low” vs “high” risk of bias |
|  | Allocation sequence concealment | Assignments could be anticipated by participants/parents and key study personnel | Allocation of participants could not be anticipated because and an acceptable method of concealment was used | Allocation concealment was not addressed or  it is unclear how allocation was concealed |
| Performance | Blinding of participants and personnel | No blinding or incomplete blinding of participants  No blinding or incomplete blinding of key study personnel | Blinding of participants ensured, and reported clearly | Unclear whether participants and/or key study personnel were blinded |
| Detection | Blinding of outcome assessment | No blinding of outcome assessors  Blinding of outcome assessors attempted but likely broken | Blinding of outcome assessors ensured and unlikely to be broken | Insufficient information to permit judgment |
| Attrition | Incomplete outcome data | Significant amount of data appears to be missing  Potentially inappropriate missing data imputation. | Complete data  Small proportion of data may be missing but the authors state clearly how they handled missing data | No description on amount of missing data  Authors do not report how missing data was handled |
| Reporting | Selective reporting | N/A | N/A | N/A |
| Other | Other bias | Study design could introduce bias  The study stopped early due to some data-dependent process  The study could be fraudulent  The study had some other problem | The study appears to be free of other type of bias | Insufficient information to assess whether an important risk of bias exists  Insufficient rationale or evidence that an identified problem will introduce bias. |

N/A: not applicable
