# Supplementary material for: Cefepime Efficacy and Safety in Children: A Systematic Review and Meta-analysis
Source: Front Pediatr. 2018 Mar 6;6:46. doi: 10.3389/fped.2018.00046 (PMC5845692; doi:10.3389/fped.2018.00046)
Supplement: Supplementary file 2 [file data_sheet_1.docx]

**Cefepime Efficacy and Safety in Children: A Systematic Review and Meta-Analysis**

Saber Jan ^1,2^, Braveen Ragunanthan ^3^, Sandra R. DiBrito ^4^, Omolabake Alabi ^5^, Maria Gutierrez ^6 *^

**Supplementary materials: Data sheet**

**1. Data extracted from each study**

The following data was extracted from each study: (1) Study characteristics : start and end date, number of participants, whether population was under 19 years of age, indication for treatment, masking [single (patient), double (patient and study personnel), triple (patient, study personnel, and outcome assessor)], whether the selected study was an open label trial, allocation ratio if specified, inclusion/exclusion criteria and type of antibiotic comparison (antibiotic versus other antibiotic, placebo or no treatment). (2) Exposure: antibiotic type, dose, frequency, route of administration, and duration of treatment. (3) Population characteristics: median, mean, and / or range of age; proportion male and female; race; body temperature and method of temperature measurement; absolute neutrophil count (ANC); hemoglobin, white blood cell count, history of recent antibiotic use; presence of documented infection; blood product infusion; history of solid tumor or hematological malignancy; receipt of granulocyte stimulating factor; presence of febrile neutropenia; receipt of chemotherapy; (4) Outcomes: mortality (as number of deaths per participants), presence of adverse events (pooled absolute number of any type), actual adverse events (as documented by the study authors), and failure of treatment (total number).

**2. Search strategy**

**PUBMED:**

("caffeine"[Supplementary Concept] OR “Atacef”[tw] OR “Anticepim”[tw] OR “Axepim”[tw] OR “Axepime”[tw] OR “Axera”[tw] OR “Bindozef”[tw] OR “Bo Shuai”[tw] OR “Bo Zhi”[tw] OR “Cefamax”[tw] OR “Cefepen”[tw] OR “Cefepime”[tw] OR “807PW4VQE3”[rn] OR "88040-23-7"[rn]OR “Cefepim”[tw] OR “cefepitax”[tw] OR “ceficad”[tw] OR “Cefinov”[tw] OR “Ceforim”[tw] OR “Cemax”[tw] OR “Cepimax”[tw] OR “Cepimex”[tw] OR “Cepim”[tw] OR “Cepiram”[tw] OR “Clocef”[tw] OR “Da Li Neng”[tw] OR “Dimipra”[tw] OR “Efipime”[tw] OR “Ekipim”[tw] OR “Forpar”[tw] OR “forzyn beta”[tw] OR “Futapim”[tw] OR “Gencef”[tw] OR “Hapimax”[tw] OR “Heng Su”[tw] OR “Imation”[tw] OR “Interpim”[tw] OR “Kai Xin”[tw] OR “Ka Luo Xin”[tw] OR “Kang Li Ou Pu”[tw] OR “Laibixin”[tw] OR “Ling Di”[tw] OR “Li Si Ping”[tw] OR “Luo Xin Wei”[tw] OR “Makenpim”[tw] OR “Maxcef”[tw] OR “Maxef”[tw] OR “maxfrom”[tw] OR “Maxicef”[tw] OR “Maxil”[tw] OR “Maximer”[tw] OR “Maxinject”[tw] OR “Maxipime”[tw] OR “Medipime”[tw] OR “Megapime”[tw] OR “Megapim”[tw] OR “Movizar”[tw] OR “Orpime”[tw] OR “Pai Nai Xin”[tw] OR “Pimcep”[tw] OR “Polyzef”[tw] OR “Pozineg”[tw] OR “Procepim”[tw] OR “Qpime”[tw] OR “Rapime”[tw] OR “Rovatim”[tw] OR “Sandocef”[tw] OR “Sanpime”[tw] OR “Sefpime”[tw] OR “Shi Rui Ping”[tw] OR “Silex”[tw] OR “Sopime”[tw] OR “Stacep”[tw] OR “Stafipime”[tw] OR “Supime”[tw] OR “Toblefam”[tw] OR “Unifepim”[tw] OR “Unisef”[tw] OR “Verapime”[tw] OR “Vipefime”[tw] OR “Xian Ke Nuo”[tw] OR “XinLiWei”[tw] OR “Ying Lan”[tw] OR “Zefipime”[tw] OR “Zepime”[tw] OR “Zepim”[tw] OR “bmy 28142”[tw] OR “bmy28142”[tw] OR “bmy-28142”[tw] OR “7 [2 (2 amino 4 thiazolyl) 2 methoxyiminoacetamido] 3 [(1 methylpyrrolidinio) methyl] 3 cephem 4 carboxylic acid”[tw] OR “7 [[(2 amino 4 thiazolyl) (methoxyimino) acetyl] amino] 3 [(1 methylpyrrolidinio) methyl] 3 cephem 4 carboxylic acid”[tw] OR “7 [alpha (2 aminothiazol 4 yl) alpha methoxyiminoacetamido] 3 [(1 methylpyrrolidinio) methyl] 3 cephem 4 carboxylic acid”[tw]) AND ((randomized controlled trial[pt] OR controlled clinical trial[pt] OR randomized[tiab] OR placebo[tiab] OR drug therapy[sh] OR randomly[tiab] OR trial[tiab] OR groups[tiab] NOT (animals [mh] NOT humans [mh]))

**EMBASE:**

('cefepime'/exp OR 'atacef':de,lnk,ab,ti,au OR 'anticepim':de,lnk,ab,ti,au OR 'axepim':de,lnk,ab,ti,au OR 'axepime':de,lnk,ab,ti,au OR 'axera':de,lnk,ab,ti,au OR 'bindozef':de,lnk,ab,ti,au OR 'bo shuai':de,lnk,ab,ti,au OR 'bo zhi':de,lnk,ab,ti,au OR 'cefamax':de,lnk,ab,ti,au OR 'cefepen':de,lnk,ab,ti,au OR 'cefepime':de,lnk,ab,ti,au OR 'cefepim':de,lnk,ab,ti,au OR 'cefepitax':de,lnk,ab,ti,au OR 'ceficad':de,lnk,ab,ti,au OR 'cefinov':de,lnk,ab,ti,au OR 'ceforim':de,lnk,ab,ti,au OR 'cemax':de,lnk,ab,ti,au OR 'cepimax':de,lnk,ab,ti,au OR 'cepimex':de,lnk,ab,ti,au OR 'cepim':de,lnk,ab,ti,au OR 'cepiram':de,lnk,ab,ti,au OR 'clocef':de,lnk,ab,ti,au OR 'da li neng':de,lnk,ab,ti,au OR 'dimipra':de,lnk,ab,ti,au OR 'efipime':de,lnk,ab,ti,au OR 'ekipim':de,lnk,ab,ti,au OR 'forpar':de,lnk,ab,ti,au OR 'forzyn beta':de,lnk,ab,ti,au OR 'futapim':de,lnk,ab,ti,au OR 'gencef':de,lnk,ab,ti,au OR 'hapimax':de,lnk,ab,ti,au OR 'heng su':de,lnk,ab,ti,au OR 'imation':de,lnk,ab,ti,au OR 'interpim':de,lnk,ab,ti,au OR 'kai xin':de,lnk,ab,ti,au OR 'ka luo xin':de,lnk,ab,ti,au OR 'kang li ou pu':de,lnk,ab,ti,au OR 'laibixin':de,lnk,ab,ti,au OR 'ling di':de,lnk,ab,ti,au OR 'li si ping':de,lnk,ab,ti,au OR 'luo xin wei':de,lnk,ab,ti,au OR 'makenpim':de,lnk,ab,ti,au OR 'maxcef':de,lnk,ab,ti,au OR 'maxef':de,lnk,ab,ti,au OR 'maxfrom':de,lnk,ab,ti,au OR 'maxicef':de,lnk,ab,ti,au OR 'maxil':de,lnk,ab,ti,au OR 'maximer':de,lnk,ab,ti,au OR 'maxinject':de,lnk,ab,ti,au OR 'maxipime':de,lnk,ab,ti,au OR 'medipime':de,lnk,ab,ti,au OR 'megapime':de,lnk,ab,ti,au OR 'megapim':de,lnk,ab,ti,au OR 'movizar':de,lnk,ab,ti,au OR 'orpime':de,lnk,ab,ti,au OR 'pai nai xin':de,lnk,ab,ti,au OR 'pimcep':de,lnk,ab,ti,au OR 'polyzef':de,lnk,ab,ti,au OR 'pozineg':de,lnk,ab,ti,au OR 'procepim':de,lnk,ab,ti,au OR 'qpime':de,lnk,ab,ti,au OR 'rapime':de,lnk,ab,ti,au OR 'rovatim':de,lnk,ab,ti,au OR 'sandocef':de,lnk,ab,ti,au OR 'sanpime':de,lnk,ab,ti,au OR 'sefpime':de,lnk,ab,ti,au OR 'shi rui ping':de,lnk,ab,ti,au OR 'silex':de,lnk,ab,ti,au OR 'sopime':de,lnk,ab,ti,au OR 'stacep':de,lnk,ab,ti,au OR 'stafipime':de,lnk,ab,ti,au OR 'supime':de,lnk,ab,ti,au OR 'toblefam':de,lnk,ab,ti,au OR 'unifepim':de,lnk,ab,ti,au OR 'unisef':de,lnk,ab,ti,au OR 'verapime':de,lnk,ab,ti,au OR 'vipefime':de,lnk,ab,ti,au OR 'xian ke nuo':de,lnk,ab,ti,au OR 'xinliwei':de,lnk,ab,ti,au OR 'ying lan':de,lnk,ab,ti,au OR 'zefipime':de,lnk,ab,ti,au OR 'zepime':de,lnk,ab,ti,au OR 'zepim':de,lnk,ab,ti,au OR '7 2 2 amino 4 thiazolyl 2 methoxyiminoacetamido 3 1 methylpyrrolidinio methyl 3 cephem 4 carboxylic acid':de,lnk,ab,ti,au OR '7 2 amino 4 thiazolyl methoxyimino acetyl amino 3 1 methylpyrrolidinio methyl 3 cephem 4 carboxylic acid':de,lnk,ab,ti,au OR '7 alpha 2 aminothiazol 4 yl alpha methoxyiminoacetamido 3 1 methylpyrrolidinio methyl 3 cephem 4 carboxylic acid':de,lnk,ab,ti,au OR 'bmy28142':de,lnk,ab,ti,au OR 'bmy 28142':de,lnk,ab,ti,au OR 'bmy-28142':de,lnk,ab,ti,au OR '807PW4VQE3' :de,lnk,ab,ti,au OR '88040-23-7':de,lnk,ab,ti,au AND ('crossover procedure':de OR 'double-blind procedure':de OR 'randomized controlled trial':de OR 'single-blind procedure':de OR random*:de,ab,ti OR factorial*:de,ab,ti OR crossover*:de,ab,ti OR (cross NEXT/1 over*):de,ab,ti OR placebo*:de,ab,ti OR (doubl* NEAR/1 blind*):de,ab,ti OR (singl* NEAR/1 blind*):de,ab,ti OR assign*:de,ab,ti OR allocat*:de,ab,ti OR volunteer*:de,ab,ti)

**CENTRAL**

(“Atacef” OR “Anticepim” OR “Axepim” OR “Axepime” OR “Axera” OR “Bindozef” OR “Bo Shuai” OR “Bo Zhi” OR “Cefamax” OR “Cefepen” OR “Cefepime” OR “Cefepim” OR “cefepitax” OR “ceficad” OR “Cefinov” OR “Ceforim” OR “Cemax” OR “Cepimax” OR “Cepimex” OR “Cepim” OR “Cepiram” OR “Clocef” OR “Da Li Neng” OR “Dimipra” OR “Efipime” OR “Ekipim” OR “Forpar” OR “forzyn beta” OR “Futapim” OR “Gencef” OR “Hapimax” OR “Heng Su” OR “Imation” OR “Interpim” OR “Kai Xin” OR “Ka Luo Xin” OR “Kang Li Ou Pu” OR “Laibixin” OR “Ling Di” OR “Li Si Ping” OR “Luo Xin Wei” OR “Makenpim” OR “Maxcef” OR “Maxef” OR “maxfrom” OR “Maxicef” OR “Maxil” OR “Maximer” OR “Maxinject” OR “Maxipime” OR “Medipime” OR “Megapime” OR “Megapim” OR “Movizar” OR “Orpime” OR “Pai Nai Xin” OR “Pimcep” OR “Polyzef” OR “Pozineg” OR “Procepim” OR “Qpime” OR “Rapime” OR “Rovatim” OR “Sandocef” OR “Sanpime” OR “Sefpime” OR “Shi Rui Ping” OR “Silex” OR “Sopime” OR “Stacep” OR “Stafipime” OR “Supime” OR “Toblefam” OR “Unifepim” OR “Unisef” OR “Verapime” OR “Vipefime” OR “Xian Ke Nuo” OR “XinLiWei” OR “Ying Lan” OR “Zefipime” OR “Zepime” OR “Zepim” OR “bmy 28142” OR “bmy28142” OR “bmy-28142” OR “7 2 2 amino 4 thiazolyl 2 methoxyiminoacetamido 3 1 methylpyrrolidinio methyl 3 cephem 4 carboxylic acid” OR “7 2 amino 4 thiazolyl methoxyimino acetyl amino 3 1 methylpyrrolidinio methyl 3 cephem 4 carboxylic acid” OR “7 alpha 2 aminothiazol 4 yl alpha methoxyiminoacetamido 3 1 methylpyrrolidinio methyl 3 cephem 4 carboxylic acid” OR "807PW4VQE3" OR "88040-23-7"):ti,ab,kw

**LILACS**

Atacef OR Anticepim OR Axepim OR Axepime OR Axera OR Bindozef OR “Bo Shuai” OR “Bo Zhi” OR Cefamax OR Cefepen OR Cefepime OR Cefepim OR cefepitax OR ceficad OR Cefinov OR Ceforim OR Cemax OR Cepimax OR Cepimex OR Cepim OR Cepiram OR Clocef OR “Da Li Neng” OR Dimipra OR Efipime OR Ekipim OR Forpar OR “forzyn beta” OR Futapim OR Gencef OR Hapimax OR “Heng Su” OR Imation OR Interpim OR “Kai Xin” OR “Ka Luo Xin” OR “Kang Li Ou Pu” OR Laibixin OR “Ling Di” OR “Li Si Ping” OR “Luo Xin Wei” OR Makenpim OR Maxcef OR Maxef OR maxfrom OR Maxicef OR Maxil OR Maximer OR Maxinject OR Maxipime OR Medipime OR Megapime OR Megapim OR Movizar OR Orpime OR “Pai Nai Xin” OR Pimcep OR Polyzef OR Pozineg OR Procepim OR Qpime OR Rapime OR Rovatim OR Sandocef OR Sanpime OR Sefpime OR “Shi Rui Ping” OR Silex OR Sopime OR Stacep OR Stafipime OR Supime OR Toblefam OR Unifepim OR Unisef OR Verapime OR Vipefime OR “Xian Ke Nuo” OR XinLiWei OR “Ying Lan” OR Zefipime OR Zepime OR Zepim OR “bmy 28142” OR bmy28142 OR bmy-28142 OR “7 2 2 amino 4 thiazolyl 2 methoxyiminoacetamido 3 1 methylpyrrolidinio methyl 3 cephem 4 carboxylic acid” OR “7 2 amino 4 thiazolyl methoxyimino acetyl amino 3 1 methylpyrrolidinio methyl 3 cephem 4 carboxylic acid” OR “7 alpha 2 aminothiazol 4 yl alpha methoxyiminoacetamido 3 1 methylpyrrolidinio methyl 3 cephem 4 carboxylic acid” OR 807PW4VQE3 OR 88040-23-7

**CLINICAL TRIALS.GOV**

“Atacef” OR “Anticepim” OR “Axepim” OR “Axepime” OR “Axera” OR “Bindozef” OR “Bo Shuai” OR “Bo Zhi” OR “Cefamax” OR “Cefepen” OR “Cefepime” OR “Cefepim” OR “cefepitax” OR “ceficad” OR “Cefinov” OR “Ceforim” OR “Cemax” OR “Cepimax” OR “Cepimex” OR “Cepim” OR “Cepiram” OR “Clocef” OR “Da Li Neng” OR “Dimipra” OR “Efipime” OR “Ekipim” OR “Forpar” OR “forzyn beta” OR “Futapim” OR “Gencef” OR “Hapimax” OR “Heng Su” OR “Imation” OR “Interpim” OR “Kai Xin” OR “Ka Luo Xin” OR “Kang Li Ou Pu” OR “Laibixin” OR “Ling Di” OR “Li Si Ping” OR “Luo Xin Wei” OR “Makenpim” OR “Maxcef” OR “Maxef” OR “maxfrom” OR “Maxicef” OR “Maxil” OR “Maximer” OR “Maxinject” OR “Maxipime” OR “Medipime” OR “Megapime” OR “Megapim” OR “Movizar” OR “Orpime” OR “Pai Nai Xin” OR “Pimcep” OR “Polyzef” OR “Pozineg” OR “Procepim” OR “Qpime” OR “Rapime” OR “Rovatim” OR “Sandocef” OR “Sanpime” OR “Sefpime” OR “Shi Rui Ping” OR “Silex” OR “Sopime” OR “Stacep” OR “Stafipime” OR “Supime” OR “Toblefam” OR “Unifepim” OR “Unisef” OR “Verapime” OR “Vipefime” OR “Xian Ke Nuo” OR “XinLiWei” OR “Ying Lan” OR “Zefipime” OR “Zepime” OR “Zepim” OR “bmy 28142” OR “bmy28142” OR “bmy-28142” OR “7 2 2 amino 4 thiazolyl 2 methoxyiminoacetamido 3 1 methylpyrrolidinio methyl 3 cephem 4 carboxylic acid” OR “7 2 amino 4 thiazolyl methoxyimino acetyl amino 3 1 methylpyrrolidinio methyl 3 cephem 4 carboxylic acid” OR “7 alpha 2 aminothiazol 4 yl alpha methoxyiminoacetamido 3 1 methylpyrrolidinio methyl 3 cephem 4 carboxylic acid” OR "807PW4VQE3" OR "88040-23-7"
